# Microfluidic preparation of size- and lamellarity-controlled liposomes using a static mixer and the advantage of a multilamellar structure in biosensing applications

**DOI:** 10.3389/fbioe.2025.1715496

**Published:** 2026-01-07

**Authors:** Makoto Kiuchi, Shinji Takeoka, Keitaro Sou

**Affiliations:** 1 Department of Life Science and Medical Bioscience, Graduate School of Advanced Science and Engineering, Waseda University, Tokyo, Japan; 2 Waseda Research Institute for Science and Engineering, Waseda University, Tokyo, Japan

**Keywords:** biosensing, fluorescent probe, immunoassay, lamellarity, liposomes, microfluidics, multilamellar, static mixer

## Abstract

**Background:**

Liposomes are useful tools for amplifying signals in biosensing techniques, which are used to detect and analyze biological molecules. The goal of this study is to improve the sensitivity of immunosorbent assays by optimizing the self-assembled structure of lipids in temperature-responsive liposomes that carry lipophilic fluorescent dyes as a detection probe in their bilayer membrane. We hypothesized that increasing the number of bilayer membranes (lamellarity) in liposomes would enhance the fluorescence signal and improve assay sensitivity, as liposomes with larger lamellarity would have a larger membrane capacity to carry the fluorescent dye per liposome of the same size.

**Methods:**

Based on this hypothesis, we first examined the microflow mixing of a lipid-ethanol solution with an aqueous medium using a static mixer to determine the optimal conditions for controlling liposomal size and lamellarity. We applied the resulting multilamellar liposomes, of which surface was modified with antibodies, as a detection probe in an immunosorbent assay for prostate-specific antigen (PSA).

**Results:**

In-line mixing with a static mixer generates liposomes of a controlled size and lamellar structure, depending on the lipid concentration, mixing ratio, and flow velocity. Notably, the initial lipid concentration in ethanol significantly impacts the lamellarity of the liposomes at a low flow velocity. The results of the immunosorbent assay using the liposomal probe indicated that a greater number of bilayer membranes produces a stronger fluorescent signal, enabling more sensitive detection of PSA.

**Conclusion:**

Static mixer-based microfluidics enables the preparation of size- and lamellarity-controlled liposomes by adjusting the processing conditions. This structural control could improve the performance of liposomes in various applications, including biosensing.

## 1 Introduction

Biosensing techniques that detect target biomolecules play a significant role in various fields, such as disease diagnosis, infectious disease control, healthcare monitoring, environmental inspections, and basic molecular biology research (Morales-Narváez and Dincer, 2020; Kabay et al., 2022). The enzyme-linked immunosorbent assay (ELISA), which combines an antigen-antibody reaction to separate the target and an enzymatic reaction to amplify the specific signal, has been used to sense target biomarkers in biological samples (Engvall and Perlmann, 1971; Peng et al., 2022; Jeon et al., 2025). The polymerase chain reaction (PCR) is a molecular biology technique that uses DNA polymerase to amplify specific DNA sequences through enzymatic replication. PCR enables the specific detection of target DNA and RNA by amplifying the signal through the replication of copies (Navarro et al., 2015; Zhu et al., 2020). These conventional biosensing techniques have significantly advanced the detection and quantification of various biomarkers. However, methods that rely on signal amplification through enzymatic reactions require a compromise between sensitivity and reaction time. Therefore, there is a need to develop biosensing techniques that can achieve both rapidity and high sensitivity in medical testing and diagnostics.

Enzyme-free biosensing techniques based on nanomaterials provide a solution to the drawbacks of conventional signal amplification using enzymatic reactions (Heydari-Bafrooei and Ensafi, 2023; Li et al., 2024). Various types of nanoparticles such as liposomes (Liu and Boyd, 2013; Ghazizadeh and Nasery, 2023), gold nanoparticles (Karnwal et al., 2024), quantum dots (Quesada-González and Merkoçi, 2025), and magnetic nanoparticles (Wang C. et al., 2024) have been used in biosensing applications. Liposomes are the extensively studied nanoparticles in the field of drug and gene delivery (Papahadjopoulos et al., 1991; Filipczak et al., 2020). Their vesicular structure allows them to encapsulate various functional molecules, including hydrophilic, hydrophobic, and amphiphilic molecules in their aqueous core and in the bilayer membrane. In addition to therapeutic agents, various types of probe molecules such as fluorescent dyes (Dong et al., 2018; Tan et al., 2019), radionuclides (Phillips et al., 2014), and contrast agents (Martina et al., 2005) can be encapsulated in liposomes for biosensing and bioimaging applications. Although the signal from a single probe molecule is weak, liposomes contribute to signal amplification because one liposome can carry thousands of probe molecules. Liposome surface can easily be customized with polymers, recognition elements such as antibodies, ligand molecules, and aptamers for specific binding with targets in biological samples and *in vivo* (Uster et al., 1996; Wang M. et al., 2024). Furthermore, the stimuli-responsive properties of liposomes can be used for the controlled release of encapsulated substances in response to stimuli, such as heat (Al-Ahmady and Kostarelos, 2016; Sarker et al., 2025). Due to their high extensibility, liposomes are expected to serve as a platform for personalized medicine based on theranostics, which combines diagnostics and therapy.

We found that liposomes with squaraine dye (SQR) in their bilayer membrane exhibit distinctive fluorescence switching properties. These properties are based on the aggregation-caused quenching and dissociation-induced emission, which occur in response to the bilayer membrane’s gel-to-liquid crystalline phase transition (Sou et al., 2019). We proposed a temperature-responsive liposome-linked immunosorbent assay (TLip-LISA) to quantify biomarkers by detecting the fluorescence switching signal (Hu et al., 2020). In the TLip-LISA, the thousands to tens of thousands of fluorescent dyes per liposome, binding with the target molecule, emit fluorescence when heated above the phase transition temperature of liposomes. The amplified temperature-responsive fluorescent signal from the liposomes can be distinguished from background noise, achieving high sensitivity. Prostate-specific antigen (PSA) and the nucleocapsid protein antigen of the SARS-CoV-2 virus have been demonstrated to be detectable by TLip-LISA (Hu et al., 2020; Hu et al., 2022). To advance biosensing techniques using liposomes, we are investigating the structural effects of liposomes on the TLip-LISA. We hypothesize that the number of bilayer membrane-forming liposomes (lamellarity) affects detection sensitivity because the degree of signal amplification should be proportional to the amount of fluorescent dye loaded per liposome. To verify this hypothesis, we explore methods for preparing liposomes with controlled lamellarity and size.

Several methods have been used for efficiently preparing size-controlled liposomes that encapsulate functional molecules. Conventional bulk methods, such as extrusion, homogenization, and ultrasonic irradiation, are top-down approaches that apply mechanical stress to lipid hydrates containing large liposomes, breaking them down into smaller, controlled-size liposomes (Mui et al., 2003; Barnadas Rodríguez and Sabés Xamaní, 2003; Lombardo and Kiselev, 2022). This approach is suitable for generating small unilamellar or a few-layered, size-controlled liposomes. However, controlling the multilamellar structure by modulating the process conditions is challenging. Microfluidic technology, through the control of rapid fluid mixing using microfluidic devices, has been studied for controlling the formation of lipid assemblies, including liposomes and lipid nanoparticles (Chen et al., 2012; Maeki et al., 2017; Cheung and Al-Jamal, 2019). Rapid mixing in microchannels enables the bottom-up approach to generate size-controlled lipid nanoparticles with a complicated inner structure of cationic or ionizable lipids and nucleic acid complexes with high reproducibility by optimizing the microchannel structure and flow conditions (Viger-Gravel et al., 2018). Recently, we proposed a microfluidic method to prepare size-controlled liposomes using a static mixer with a scalable in-line element in a channel (Ota et al., 2023). Previous research revealed that the microfluidics method using a static mixer could control the lamellarity of liposomes, in addition to their size, by adjusting the microfluidic conditions. This paper systematically studies the microfluidic conditions for controlling the size and lamellarity of liposomes using a static mixer. Then, we evaluated the effect of the lamellarity of liposomes on biomarker detection sensitivity by comparing liposomes with different lamellarity. The results demonstrated that liposomal probes with higher lamellarity are more sensitive to detecting biomarkers.

## 2 Materials and methods

### 2.1 Materials

1,2-Dipalmitoyl-*sn*-glycero-3-phosphocholine (DPPC) and cholesterol were purchased from Tokyo Chemical Industry Co., Ltd. (Tokyo, Japan). 1,5-Dihexadecyl-*N*-succinyl-L-glutamate (DHSG) was synthesized in-house according to the previously published procedure (Sou et al., 2003). *N*-(Methylpolyoxyethylene oxycarbonyl)-1,2-distearoyl-*sn*-glycero-3-phosphoethanolamine, sodium salt SUNBRIGHT DSPE-050CN (PEG-DSPE) and *N*-[(3-maleimide-1-oxopropyl)aminopropyl-polyethyleneglycol-carbamyl]-distearoylphosphatidyl-ethanolamine SUNBRIGHT DSPE-020MA (MAL-PEG-DSPE) were purchased from NOF Co. (Tokyo, Japan). 1,2-Distearoyl-*sn*-glycero-3-phosphoethanolamine-*N*-[biotinyl (polyethylene glycol)-2000] (Biotin-PEG-DSPE) was purchased from Avanti Polar Lipids (Alabaster, AL). Magnetic particles (Magnosphere MS300/Streptavidin and Magnosphere MS300/Carboxyl) were purchased from JSR Life Sciences (Tokyo, Japan). Anti-human prostate-specific antigen (PSA) monoclonal antibodies (clone: 4D10 for capture and clone: 56 for detection) were purchased from Mikuri Immunology Laboratory (Osaka, Japan). PSA from human semen was purchased from Sigma-Aldrich (St. Louis, MO, United States). The synthesis and characterization of the fluorescent dye (2-[4-(dibutylamino)phenyl]-4-[(3,3-dimethyl-3*H*-indol-2-yl)methylene]-3-hydroxycyclobut-2-en-1-one, SQR22) have been reported previously (Sou et al., 2019). SQR22 was obtained by custom synthesis from the NARD Institute (Kobe, Japan).

### 2.2 Experimental design

**FIGURE 1:**
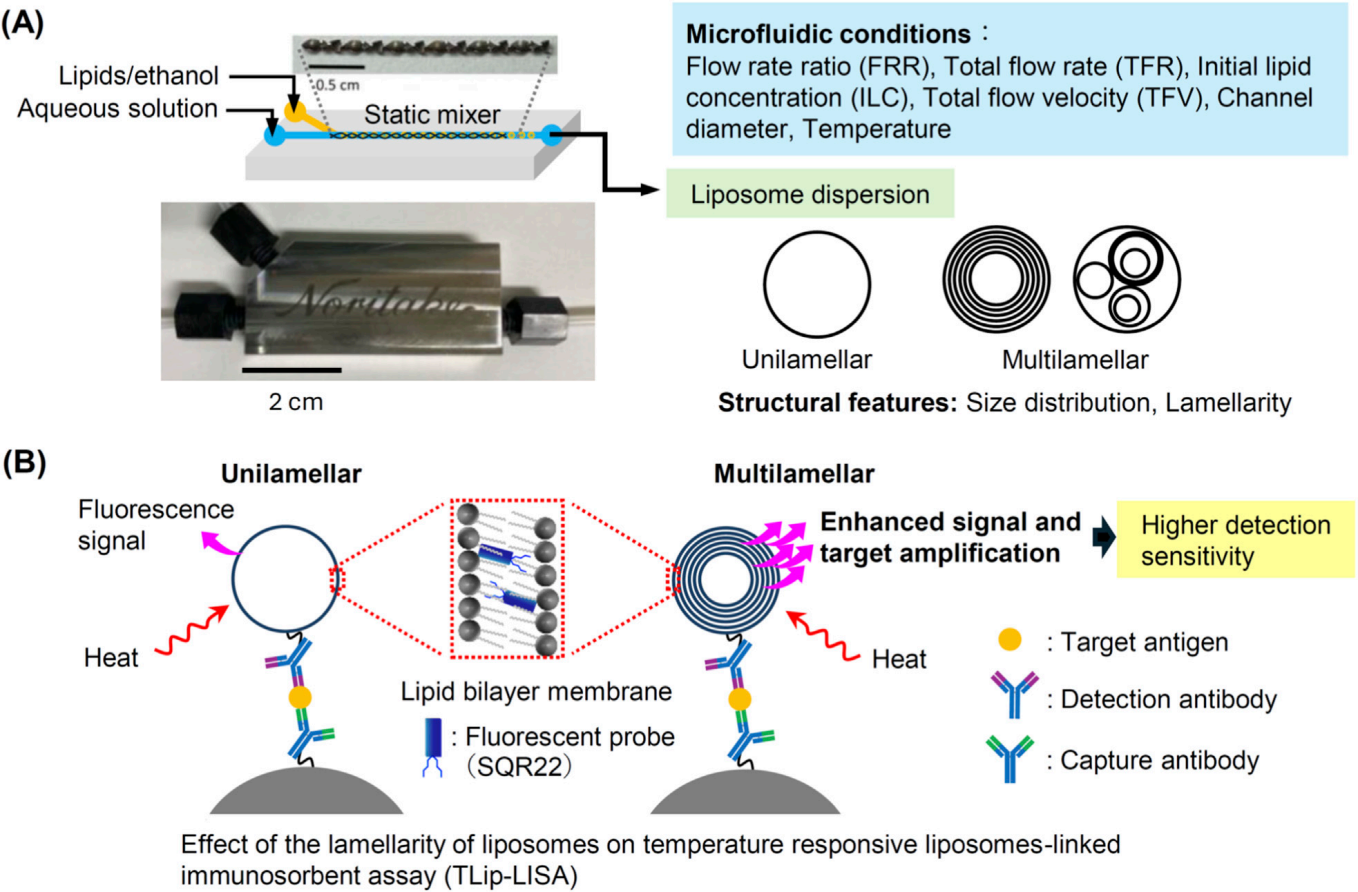
Schematic of the concept and experimental design of this study. **(A)** Microfluidic preparation of liposomes using a static mixer. The size and lamellarity of liposomes are controlled by adjusting the microfluidic conditions. **(B)** A schematic diagram showing how the temperature-responsive liposomal fluorescent probes detect the target antigen. This study focuses on how the lamellarity of liposomes affects target detection sensitivity under the hypothesis that signal amplification is enhanced by increasing the number of bilayer membranes in liposomes of the same size.

Figure 1 illustrates the conceptual design of this study. First, we investigate microfluidic conditions such as the flow rate ratio (FRR), the total flow rate (TFR), the initial lipid concentration (ILC), the channel diameter, the total flow velocity (TFV), and the temperature to control the size and lamellarity of liposomes, as shown in Figure 1A. The liposomes obtained under various conditions are characterized by their particle size and lamellarity. The microfluidic conditions to obtain multilamellar liposomes will be applied to prepare temperature-responsive immunoliposomes embedding a fluorescence probe molecule (SQR22) in the bilayer membrane. For reference, temperature-responsive immunoliposomes embedding SQR22 will be prepared using the conventional extrusion method. These liposomes will then be used as immunofluorescent probes in a temperature-responsive liposome-linked immunosorbent assay (TLip-LISA) to detect PSA, and the limit of detection will be compared to evaluate the effect of lamellarity of liposomes on the detection sensitivity (Figure 1B).

### 2.3 Preparation of liposomes with a static mixer

Liposomes were prepared using a static mixer (SM) under various conditions. The SM used was a K-R type SM (K1-24R-3, with diameters of 0.6 or 1.0 mm, SUS) provided by Noritake (Aichi, Japan). DPPC, cholesterol, DHSG, and PEG-DSPE were dissolved in t-butyl alcohol at a molar ratio of 5:5:1:0.066. The solution was then freeze-dried to obtain a stock of mixed lipid powder. An aliquot of the freeze-dried powder was dissolved in 99.5% ethanol (Fujifilm Wako Pure Chemical Co., Japan). The initial lipid concentration (ILC) was set to 30, 60, 90, and 120 mg/mL in ethanol. A syringe pump (Legato 111, KD Scientific, United States) was used to pass the lipid-ethanol solution and the aqueous solution (Dulbecco’s phosphate buffered saline, DPBS) containing 0.02 wt% sodium azide (DPBS [0.02% NaN_3_]) through the SM device. The total flow velocity (TFV) was set between 3.2 and 70.7 cm/s. The aqueous-to-ethanol flow rate ratio (FRR) was set between 2 and 9.

The dispersion medium of the obtained liposome dispersion was replaced via centrifugal ultrafiltration using a Vivaspin® 6 with a 100,000-MWCO filter to remove the ethanol. The liposome dispersion was then concentrated to one-tenth of the original volume in a Vivaspin® 6 by centrifugation (10,000 rpm for 20–25 min) at 4 °C. Fresh DPBS (0.02% NaN_3_) was then added to the concentrated liposome dispersion to reach the original volume. This cycle of concentrating and diluting the solution 10-fold with DPBS (0.02% NaN_3_) was repeated three times. The final ethanol concentration is theoretically estimated to be reduced to 1/1000.

### 2.4 Preparation of biotin-conjugated temperature-responsive liposomes

Biotin-conjugated temperature-responsive liposomes were prepared using a K-R type SM (K1-24R-3, 1.0 mmφ, SUS). DPPC, DHSG, and Biotin-PEG-DSPE were dissolved in t-butyl alcohol at a molar ratio of 86.6:9.6:0.5. The solution was then freeze-dried to obtain a stock of mixed lipid powder. An aliquot of the freeze-dried powder was dissolved at 30, 60, and 90 mg/mL in 99.5% ethanol (Fujifilm Wako Pure Chemical, Japan), along with or without 3.3 mol% SQR22 relative to total lipids. The lipid-ethanol solution and DPBS (0.02% NaN_3_) were passed through the SM device at TFR of 1.5 mL/min (TFV of 3.2 cm/s) and aqueous-to-ethanol FRR of 3. The TFR and FRR were adjusted using two syringe pumps (Legato 111, KD Scientific, United States). The processing temperature was controlled by immersing the connecting tubes filled with the solutions and the SM device in a water bath set at 50 °C. The ethanol in the obtained liposome dispersion was removed by centrifugal ultrafiltration using a Vivaspin® 6, as described in Section 2.3.

A sample of unilamellar biotin-conjugated temperature-responsive liposomes with the same lipid composition and SQR22 was prepared using the hydration/extrusion method. The mixed lipid powder was hydrated at 8 mg/mL in DPBS (0.02% NaN_3_) by stirring with a stirrer bar (600 rpm) at 50 °C overnight. After hydration, the mixture was passed through membrane filters with pore sizes of 0.2 μm and 0.05 μm three times each using an extruder (LIPEX, Northern Lipids Inc., Vancouver, Canada) at 58 °C.

### 2.5 Preparation of IgG-conjugated temperature-responsive liposomes

#### 2.5.1 Preparation of maleimide-modified temperature-responsive liposomes (Mal-TLip)

Temperature-responsive liposomes were prepared using a K-R type SM (K1-24R-3, 1.0 mmφ, SUS). The lipids, along with SQR22 (DPPC, DHSG, and SQR22 at a molar ratio of 86.6:9.6:3.3), were dissolved in t-butyl alcohol. The solution was then freeze-dried to obtain a stock of mixed lipid powder. An aliquot of the freeze-dried powder was dissolved at 90 mg/mL in 99.5% ethanol (Fujifilm Wako Pure Chemical, Japan). The lipid-ethanol solution and DPBS (0.02% NaN_3_) were passed through the SM device at TFR of 1.5 mL/min (TFV of 3.2 cm/s) and aqueous-to-ethanol FRR of 3, which were adjusted using two syringe pumps (Legato 111, KD Scientific, United States). The processing temperature was controlled by immersing the connecting tubes filled with the solutions and the SM device in a water bath set at 55 °C. The ethanol in the obtained liposome dispersion was removed by centrifugal ultrafiltration using a Vivaspin® 6, as described in Section 2.3.

For the liposome sample prepared by the hydration and extrusion method, an aliquot of the freeze-dried mixed lipids (DPPC, DHSG, and SQR22 at a molar ratio of 86.6:9.6:3.3) was hydrated with 2 mL of DPBS at a lipid concentration of 4 mg/mL. After vortex mixing for 15 min, the dispersion was extruded twice through a 25 mm, 0.2 μm pore-sized, track-etched polycarbonate membrane (Nucleopore, Whatman, Maidstone, United Kingdom) using an extruder (LIPEX, Northern Lipids Inc., Vancouver, Canada) pre-heated to 58 °C.

Maleimide-PEG-DSPE was incorporated into the prepared temperature-responsive liposomes. An ethanol solution of maleimide-PEG-DSPE (10 mg/mL) was added to the liposome dispersion. The amount of maleimide-PEG-DSPE added was calculated based on the lamellarity of the liposomes and adjusted to 0.37 mol% of the lipids in the outermost layer. The lipid amount in the outermost layer was calculated as (total lipid amount/lamellarity)/2 under the assumption that the amount of lipids composing each lipid layer is the same as the amount of lipids composing the outermost layer of liposomes. The mixture was then incubated at 37 °C for 30 min while stirring with a stirrer bar at 600 rpm. After incubation, the mixture was ultracentrifuged at 50,000 g for 40 min. The supernatant, which contained the unincorporated maleimide-PEG-DSPE, was removed. The precipitated maleimide-modified temperature-responsive liposomes (Mal-TLip) were collected and redispersed in DPBS (0.02% NaN_3_). The concentration was adjusted to 2 mg/mL as the lipid concentration of the outermost layer of the liposome, which was calculated as (total lipid concentration/lamellarity)/2 under the assumption that the amount of lipids composing each lipid layer is the same as the amount of lipids composing the outermost layer of liposomes.

#### 2.5.2 Preparation of the 2-iminothiolane-modified antibody (IgG-SH)

First, Traut’s solvent (25 mM HEPES, 140 mM sodium chloride, and 3 mM EDTA), HBS (140 mM sodium chloride, 1.5 mM sodium dihydrogen phosphate, and 50 mM HEPES), and a 0.123 mg/mL solution of 2-iminothiolane hydrochloride (dissolved in Traut’s solvent) were prepared. To prepare the 2-iminothiolane-modified antibody, 200 mg of anti-human PSA monoclonal antibody (clone: 56) was mixed with a 2-iminothiolane hydrochloride solution at a molar ratio of 1:20. The mixture was incubated at room temperature for 1 h with gentle shaking. Then, 2 mL of HBS was added to the mixture, which was loaded onto a 50 kDa Amicon Ultra centrifugal filter device (Merck Millipore, Burlington, United States) and centrifuged at 10 °C, 15,000 g for 10 min to remove the unreacted 2-iminothiolane hydrochloride. A spectrophotometer was used to analyze the concentration of the product at a wavelength of 280 nm. The concentration of IgG-SH was calculated from the absorbance measured at 280 nm with a spectrophotometer, using an extinction coefficient of 1.38 mL mg^-1^·cm^-1^.

#### 2.5.3 Conjugation of Mal-TLip and IgG-SH

The Mal-TLip dispersion (the lipid concentration in the outermost layer of liposomes is 2 mg/mL, 1 mL) was mixed with 200 μL of the IgG-SH solution (with a concentration of 0.7 mg/mL) and incubated overnight at room temperature with gentle shaking. Then, 30 μL of the 0.8 mM N-acetylcysteine solution was added to the reaction mixture to block the unreacted maleimide group. The mixture was then incubated at room temperature for 10 min. Ultrafiltration was then performed on the final product using a 100 kDa Vivaspin® 20 centrifugal ultrafiltration device (Sartorius, Göttingen, Germany) at 10 °C for 10 min at 15,000 g to remove unconjugated IgG-SH and other unreacted molecules. The obtained dispersion of pure IgG-TLip was stored in a refrigerator under dark conditions.

### 2.6 Characterization of liposomes and IgG-TLip

The size distribution, mean diameter, and zeta potential of the prepared liposomes were measured by the Zetasizer Nano S90 (Malvern Instruments Ltd., Malvern, United Kingdom) using the dynamic light scattering technique. The concentration of lipids in the liposome samples was determined using a phospholipid assay kit based on an enzymatic method using choline oxidase, *N*-ethyl-*N*-(2-hydroxy-3-sulfopropyl)-3,5-dimethoxyaniline, and 4-aminoantipyrine (Fujifilm Wako Pure Chemical, Japan). To determine the concentration of SQR22 in the liposome samples, a standard curve of SQR22 was obtained from the absorbance measured at 631 nm with different concentrations of SQR22 ethanol solution. An aliquot (20 µL) of the liposome dispersion was mixed with 1 mL of ethanol, and the absorbance of the solution was measured at 631 nm. The concentration of SQR22 in the liposome samples was then determined based on the standard curve.

To determine the lamellarity of liposomes, we used a fluorescence probe, 6-*p*-toluidino-2-naphthalenesulfonic acid (TNS) (Abcam, United Kingdom), which emits fluorescence upon adsorbing to the surface of liposomes. The fluorescence intensity is proportional to the total surface area of liposomes. Therefore, the number of bilayer membranes (lamellarity) can be calculated by comparing the fluorescence intensity with that of standard unilamellar liposomes at the same lipid concentration (Takeoka et al., 1996). TNS was added to liposome samples with a three-step lipid concentration gradient and incubated at ambient temperature for 12 h before measuring fluorescence intensity (λ_ex_ = 321 nm, λ_em_ = 400 nm). After measuring the fluorescence intensity, the relationship between the amount of liposome sample added and the fluorescence intensity was plotted on a graph, and the slopes (*S*_*b*_) were calculated (Supplementary Figure S1). The number of bilayer membranes of liposomes (lamellarity) was calculated by dividing the slope of the standard unilamellar liposomes prepared by the extrusion method (*S*_*a*_) under the assumption that the number of lipids composing each lipid layer is the same as the number of lipids composing the outermost layer of liposomes (Supplementary Table S2) from Equation 1.

$$Lamellarity=S_{a}/S_{b}$$ (1)

The lamellarity of liposomes containing SQR22 was determined from the relationship between lamellarity and the turbidity of liposome dispersions to avoid the interference from SQR22 in the fluorescence measurements (Supplementary Figure S2). A calibration curve between lamellarity, as determined by the TNS method described above, and turbidity, as quantified by the absorbance at 800 nm ([lipids] = 0.5 mg/mL), was created using liposomes prepared without SQR22 at the same lipid composition and preparation conditions. The absorbance at 800 nm was measured for liposomes containing SQR22 ([lipids] = 0.5 mg/mL), and lamellarity was calculated from the calibration curve.

The fluorescamine assay was used to determine the concentration of IgG in the IgG-TLip dispersion. The Mal-TLip was diluted to an SQR22 concentration of 10 μg/mL. Then, 20 µL of the IgG-SH samples with different concentrations were mixed with 20 µL of the Mal-TLip samples to prepare a standard solution of IgG in the presence of Mal-TLip with an SQR22 concentration of 5 μg/mL. The IgG-TLip sample was diluted with DPBS to an SQR22 concentration of 5 μg/mL. Next, 40 μL of the standard and sample solutions were mixed with 40 μL of OG and incubated in a water bath at 50 °C for 30 min. After incubation, 300 μL of borate buffer solution (pH 9.0) was added to the solutions and mixed by vortexing. Then, 300 μL of a 1 mg/mL fluorescamine solution was added to the solutions. After further vortex mixing, the mixture was incubated in a dark chamber at room temperature for 10 min. Then, 180 μL of the standard or sample solution mixtures were applied to a black 96-well plate, and the fluorescence intensity was measured (λ_ex_ = 381 nm, λ_em_ = 476 nm). The fluorescence data from the standard solution were plotted against the IgG concentration to create a calibration curve. The IgG concentration in the original IgG-TLip sample was then determined using the calibration curve.

### 2.7 Comparison of the fluorescent signal amplification by TLips with various lamellarities

A magnetic microparticle modified with streptavidin (Magnosphere™ MS300/Streptavidin, Stv-MMP) was used to capture biotin-TLip with different lamellarity in order to compare the fluorescence signal intensity. Biotin-TLip with SQR22 dispersion ([lipids] = 5.0 mg/mL, 100 μL) and Stv-MMP dispersion ([solid] = 0.1 mg/mL, 100 μL) were mixed in a microtube and shaken at room temperature for 10 min. After the reaction, the Stv-MMP was collected at the bottom of the microtube using a neodymium magnet and washed twice with 200 μL of DPBS to remove the unreacted biotin-TLip. The Stv-MMP was dispersed in 200 μL of DPBS again and added in 100 μL portions to 1 × 8 stripwell plates. The bottom of the well was heated on a hot plate set at 80 °C while the change in fluorescence intensity before and after heating was measured using a fluorescence detector (FLE1100) with a micro-optic probe (Probe40100) (Nippon Sheet Glass, Kanagawa, Japan) connected to a computer. Three measurements were performed for each Biotin-TLip sample, and the results were presented as averages with standard deviations.

### 2.8 Detection of PSA by using IgG-TLip and IgG-MMP

Anti-human PSA antibody-conjugated magnetic microparticles (IgG-MMP) were prepared via chemical crosslinking between the amino group of the capture antibody and the carboxyl group on the magnetic microparticles (Magnosphere™ MS300/Carboxyl, Carboxyl-MMP) (See Supplementary Material for details). An IgG-MMP dispersion with a solid concentration of 100 μg/mL ([IgG] = 0.631 μg/mL, 100 μL) was mixed with 100 μL of a PSA standard solution with concentrations of 0 (negative control), 1, 10, 100 fg/mL, 1, 10, 100 pg/mL in a microtube. The mixture was shaken at room temperature for 1 min. The IgG-MMP was collected at the bottom of the microtube using a neodymium magnet. The supernatant was removed, and the unbound PSA was removed by washing twice with 100 μL of DPBS. Then, IgG-TLip dispersion ([IgG] = 0.5 μg/mL, 100 μL) was added to the microtubes. The tubes were shaken at room temperature for 1 min, and the IgG-MMP was collected using a neodymium magnet. The supernatant was removed, and the unbound IgG-TLip was removed by washing twice with 100 μL of DPBS. The mixture was dispersed in 200 μL of DPBS again and added in 100 μL portions to 1 × 8 stripwell plates. The bottom of the well was heated on a hot plate set at 80 °C while the change in fluorescence intensity before and after heating was measured using a fluorescence detector (FLE1100) with a micro-optic probe (Probe40100) (Nippon Sheet Glass, Kanagawa, Japan) connected to a computer. Three measurements were performed for each IgG-TLip sample at each PSA concentration, and the results were presented as averages with standard deviations. The limit of detection was determined as the PSA concentration corresponding to the fluorescence intensity equal to the average fluorescence intensity of the negative control plus three times the standard deviation of the negative control in the fitting curve between PSA concentration and fluorescence intensity.

## 3 Results

### 3.1 Structural control of liposomes using a static mixer

**FIGURE 2:**
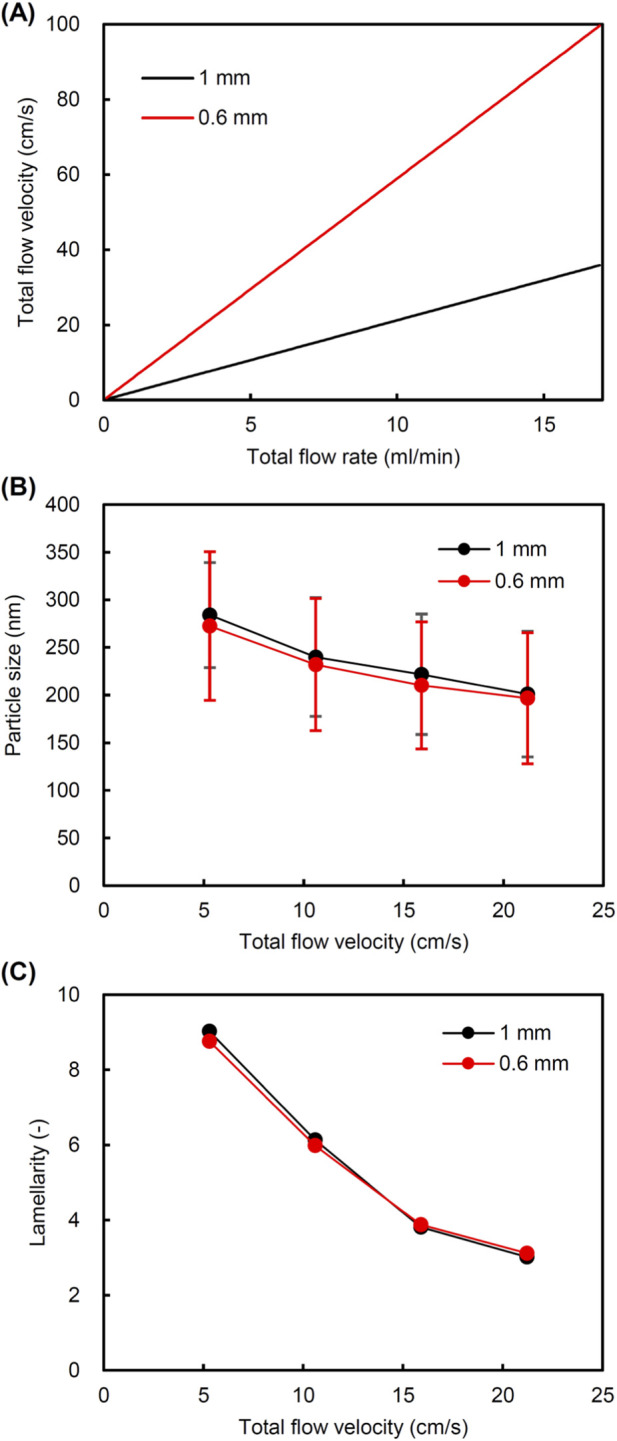
Comparison of microfluidic preparation of liposomes using static mixers with different channel diameter. **(A)** Relationship between total flow rate (TFR) and total flow velocity (TFV) of fluids flowing through a channel with a diameter of 0.6 mm or 1 mm; **(B)** particle size of liposomes prepared using a static mixer with a 0.6 mm or 1 mm channel; and **(C)** lamellarity of liposomes prepared using a static mixer with a 0.6 mm or 1 mm channel.

This study used two types of static mixers with channel diameters of 0.6 mm and 1 mm. The detachable element consists of rectangular plate units that are twisted at 180° and connected in alternating right and left twists (Ota et al., 2023). The elements have the same diameter as the channel, and the pitch of the unit in the flow direction is 1.5 mm. The cross-sectional area of the 1 mm channel is 2.8 times larger than that of the 0.6 mm channel. Therefore, the TFVs were different, even when the TFR is the same (Figure 2A). To verify whether static mixers with different channel size have comparable mixing efficiency at the same TFV, we compared the characteristics of liposomes prepared using two static mixers at the same TFV. As shown in Figures 2B,C, the particle size and lamellarity of liposomes are equal between the two static mixers when compared with the same TFV. The liposome size and lamellarity decreased as the TFV increased, showing the same trend. These results indicate that the mixing efficiency depends on the TFV regardless of the channel diameter. Therefore, the flow conditions are represented as a TFV rather than a TFR to compensate for the difference in the channel diameter. We varied the TFV over a wide range by using static mixers with channel diameters of 0.6 mm and 1 mm.

**FIGURE 3:**
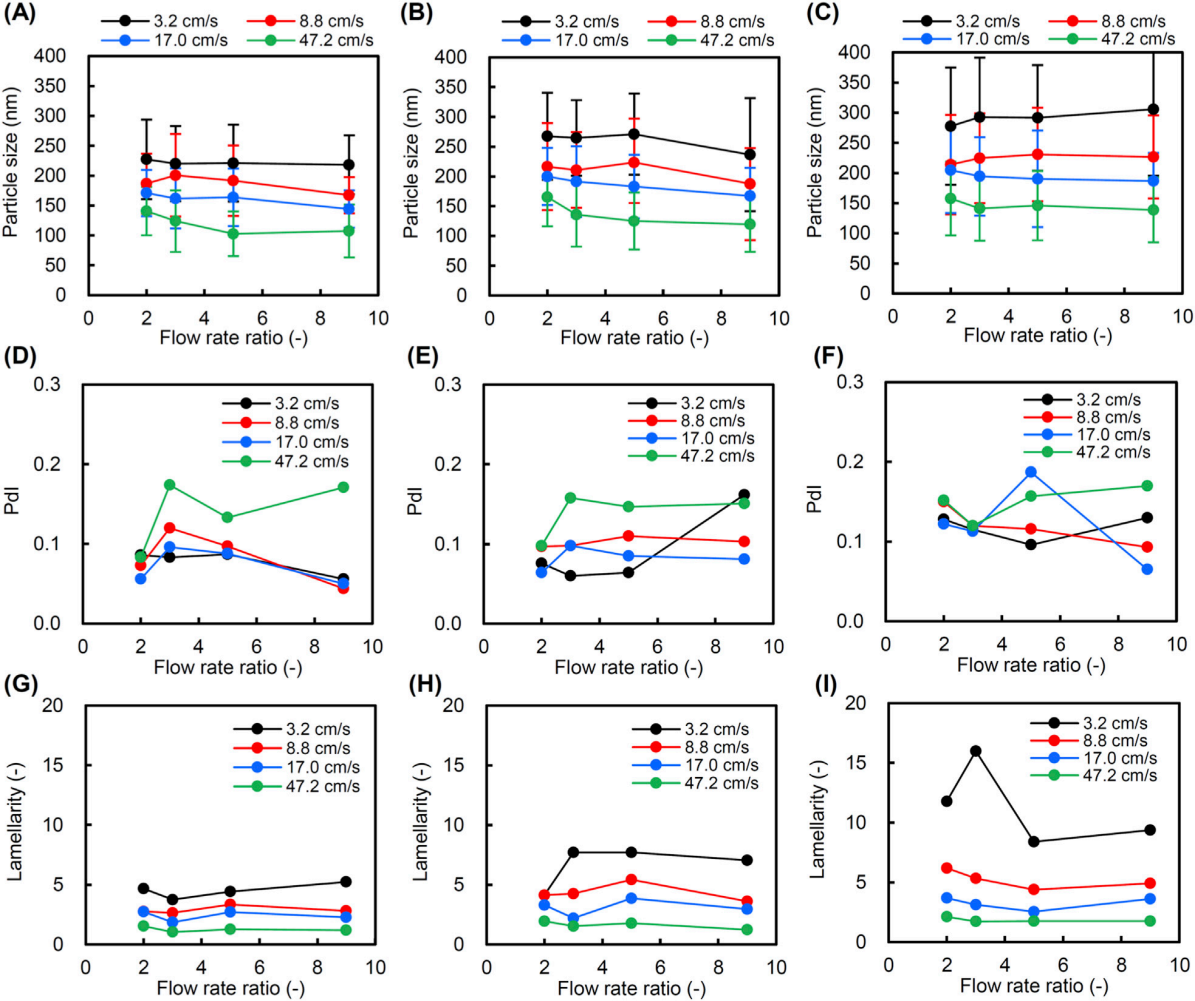
Size and lamellarity of liposomes prepared using a static mixer at various flow rate ratios (FRRs) of aqueous and lipid/ethanol solutions at ambient temperature. Four total flow velocities (TFVs) of 3.2, 8.8, 17.0, and 47.2 cm/s were applied at each FRR. **(A)** Particle size at an initial lipid concentration (ILC) of 30 mg/mL, **(B)** particle size at an ILC of 60 mg/mL, **(C)** particle size at an IL of 90 mg/mL, **(D)** polydispersity index (PdI) at an ILC of 30 mg/mL, **(E)** PdI at an ILC of 60 mg/mL, **(F)** PdI at an ILC of 90 mg/mL, **(G)** lamellarity at an ILC of 30 mg/mL, **(H)** lamellarity at an ILC of 60 mg/mL, and **(I)** lamellarity at an ILC of 90 mg/mL.

Next, we investigated the effect of the FRR by changing the ratio of the aqueous solution to the lipid/ethanol solution from 2:1 to 9:1. As shown in Figures 3A–C, the particle size of liposomes did not change significantly when the FRR was varied at the same TFV. On the other hand, the particle size decreased as the TFV increased in the same FRR at all ILCs. The polydispersity index (PdI), which represents the degree of size distribution, was less than 0.2 for all tested conditions (Figures 3D–F). There was no significant change in the lamellarity of liposomes when the FRR was changed from 2 to 9 at the ILC of 30 mg/mL (Figure 3G). On the other hand, the lamellarity of liposomes increased when prepared at the FRR of 3 and the TFV of 3.2 cm/s at the ILCs of 60 and 90 mg/mL (Figures 3H,I). However, this trend was not observed at the TFV of 8.8 cm/s or above. The lamellarity decreased as the TFV increased in the same FRR at all ILCs.

**FIGURE 4:**
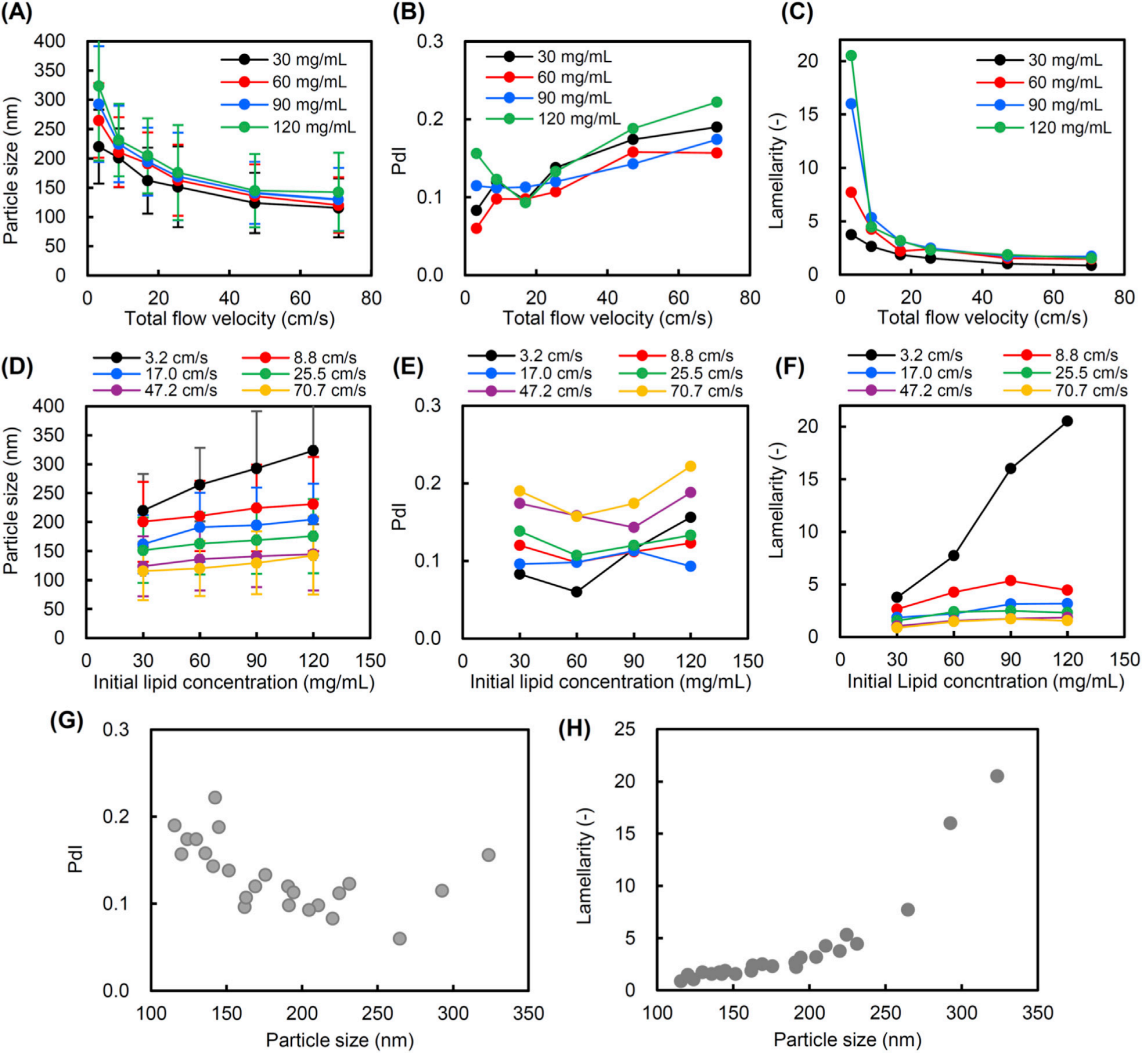
Size and lamellarity of liposomes prepared using a static mixer at various total flow velocities (TFVs) and initial lipid concentrations (ILCs) at ambient temperature. The flow rate ratio (FRR) of aqueous and lipid/ethanol solutions was fixed at 3. **(A)** Particle size, **(B)** polydispersity index (PdI), and **(C)** lamellarity as a function of TFV. **(D)** Particle size, **(E)** PdI, and **(F)** lamellarity as a function of ILC. **(G)** Scatter plot of particle size and PdI of liposomes prepared using a static mixer under various conditions. **(H)** Scatter plot of particle size and lamellarity of liposomes prepared using a static mixer under various conditions.

**FIGURE 5:**
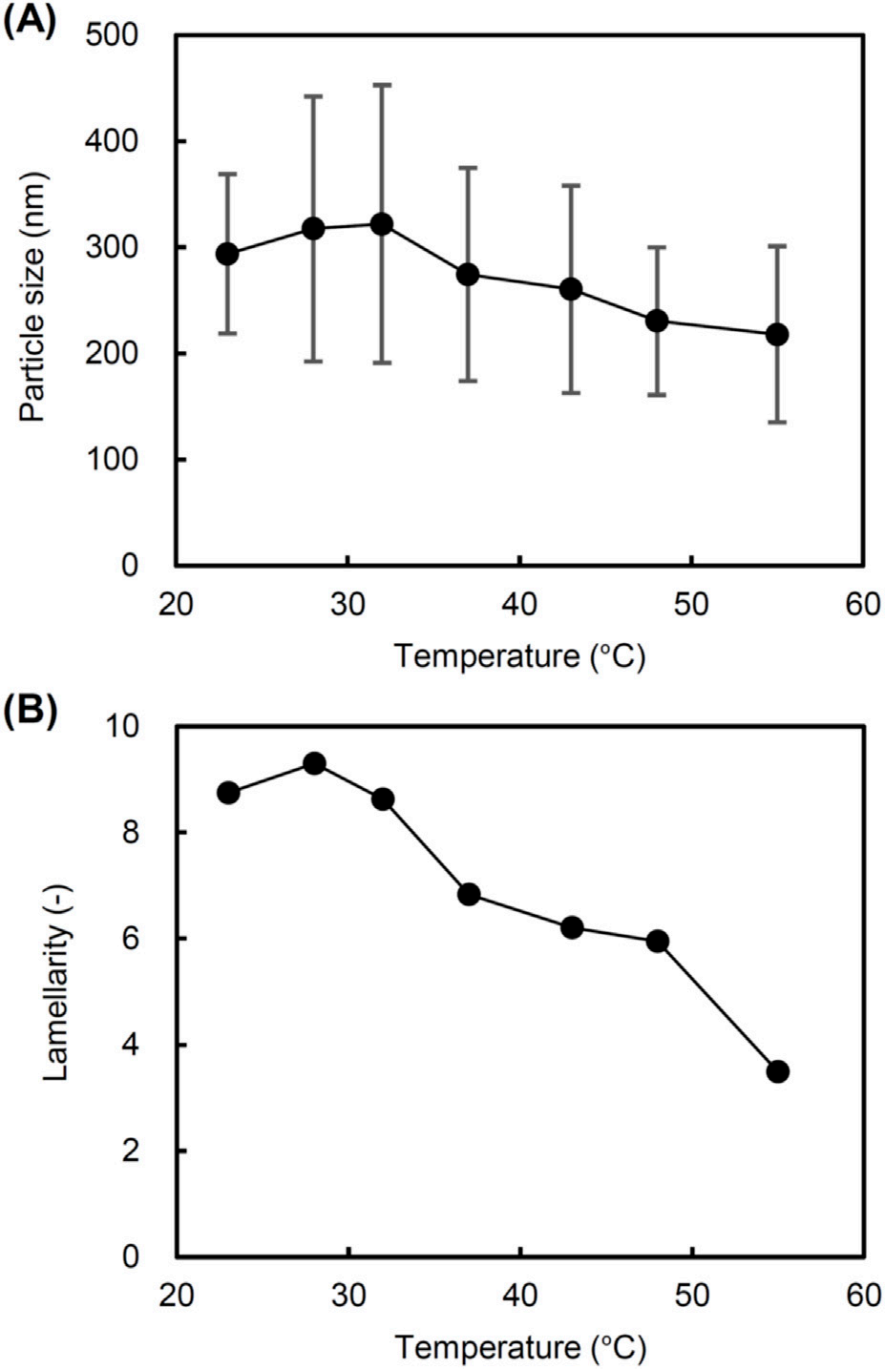
Size and lamellarity of liposomes prepared using a static mixer at various temperatures. The following parameters were used: a total flow velocity (TFV) of 3.2 cm/s; a flow rate ratio (FRR) of 3, and an initial lipid concentration (ILC) of 90 mg/mL. **(A)** Particle size and **(B)** lamellarity.

The particle size of liposomes decreased as the TFV increased from 3.2 to 70.7 cm/s at all ILCs (Figure 4A). The PdI tends to increase with increasing TFV (Figure 4B). Lamellarity decreased significantly with increasing TFV below 25.5 cm/s (Figure 4C). The variation in lamellarity was particularly large at ILCs of 90 and 120 mg/mL in low TFV. Regardless of the ILC, the change in lamellarity was slight at the TFV of 25.5 cm/s or higher. To recognize the correlation with ILC, we plotted the particle size, PdI, and the lamellarity of liposome against the ILC. The particle size of liposomes increased with increasing ILC at any TFV. In particular, the size variation by changing the ILC was most pronounced at the lowest TFV of 3.2 cm/s (Figure 4D). A clear correlation between ILC and PdI was not observed (Figure 4E). Lamellarity tended to increase with increasing ILC, and this correlation was particularly significant at the TFV of 3.2 cm/s (Figure 4F). Figures 4G,H are scatter plots showing the relationships between the particle size and PdI, and between the particle size and lamellarity of liposomes prepared under various conditions. The mean particle size was changed in a range from 115 to 324 nm with a narrow size distribution (PdI of 0.2 or less) (Figure 4G). In particular, the PdIs were as low as 0.1 for particle sizes between 150 and 300 nm, indicating that the size of liposomes can be controlled with a narrow distribution in this size range. The scatter plot of size versus lamellarity shows that liposomes with larger particle sizes have higher lamellarity (Figure 4H). Furthermore, we studied the influence of temperature on the structural control of liposomes using a static mixer. As shown in Figures 5A,B, the particle size and lamellarity decreased as the temperature increased above 32 °C.

### 3.2 Effect of lamellarity of liposomes on the fluorescent signal amplification

**FIGURE 6:**
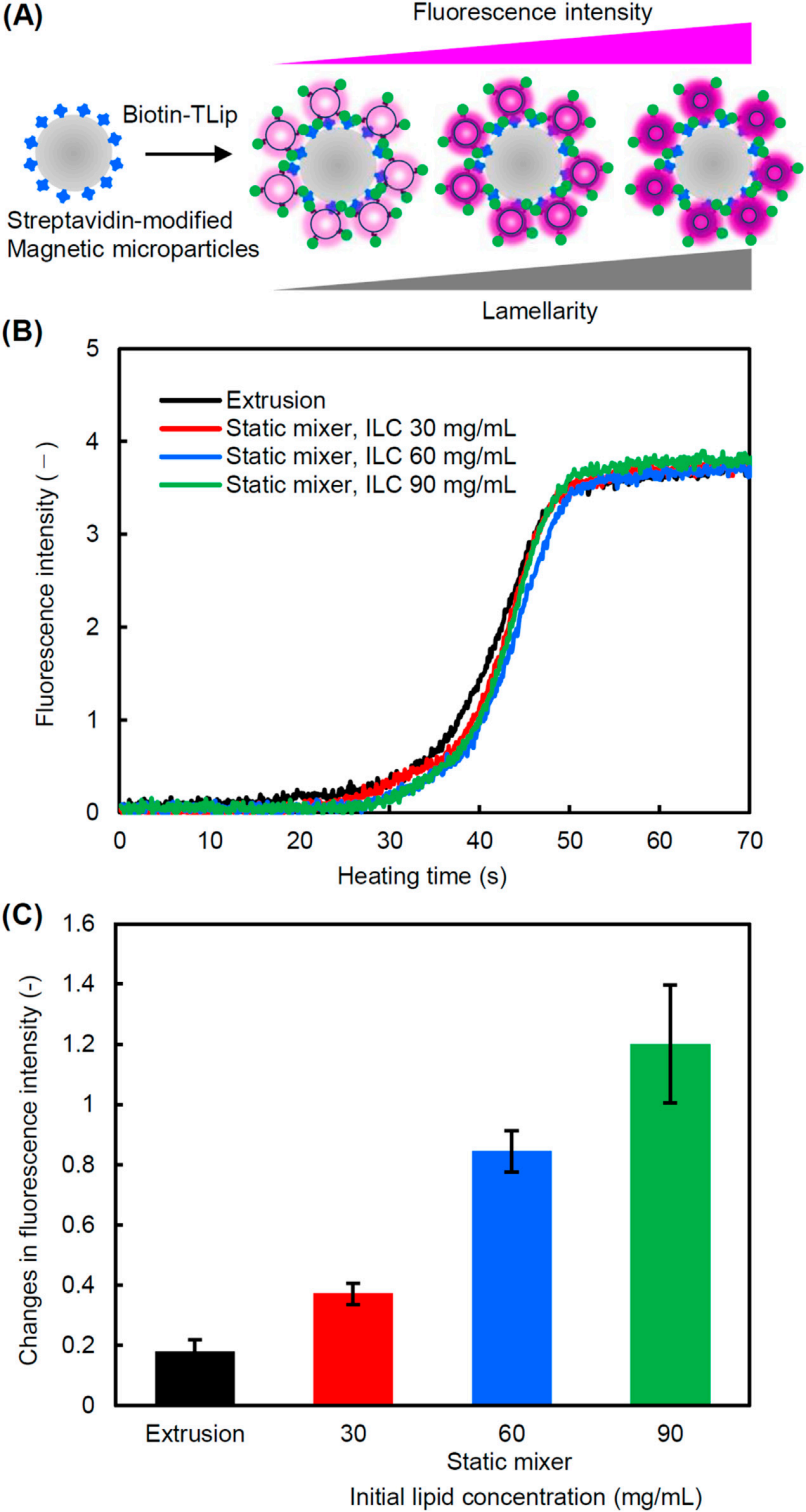
Effect of lamellarity of liposomes on the fluorescence signal intensity. **(A)** Schematic diagram showing the correlation between fluorescence intensity and lamellarity of Biotin-TLip captured by streptavidin-modified magnetic microparticles. **(B)** Profiles of fluorescence intensity changes upon heating of Biotin-TLip dispersions ([lipid] = 0.1 mg/mL), **(C)** Changes in fluorescence intensity of Biotin-TLip binding to streptavidin-modified magnetic microparticles by heating (n = 3).

To verify the hypothesis that the higher lamellarity in liposomes results in greater signal amplification (Figure 6A), we prepared several biotin-modified temperature-responsive liposomes (Biotin-TLip) with varying lamellarity by adjusting the ILC in preparation using a static mixer (Table 1). Liposomes prepared by an extrusion method with a final filter pore size of 0.05 µm were used as unilamellar liposomes for comparison. First, we compared the change in fluorescence intensity of Biotin-TLip with different lamellarity in a dispersion state at the same SQR22 concentration by heating (Figure 6B). The fluorescence intensity of the Biotin-TLip dispersion increased around 30 s after heating it on a hot plate, indicating that the temperature of the dispersion had partially reached the phase transition temperature of the Biotin-TLip (41 °C). The fluorescence intensity reached a plateau around 50 s, indicating that all Biotin-TLip had completed transition from the gel phase to the liquid crystal phase. There are no notable differences in the profiles of the change in fluorescence intensity due to the difference in the lamellarity of Biotin-TLip.

**TABLE 1:** Characterization of biotin-modified temperature-responsive liposomes (Biotin-TLip).

Preparation method	Lamellarity (−)	Particle size (nm)	PdI
Extrusion (0.05 µm)	1.0	102 ± 31	0.09
Static mixer, ILC: 30 mg/mL	1.4	190 ± 64	0.17
Static mixer, ILC: 60 mg/mL	4.5	210 ± 68	0.17
Static mixer, ILC: 90 mg/mL	7.5	226 ± 95	0.21

Next, the Biotin-TLip dispersions were mixed with streptavidin-modified magnetic microparticles to capture the TLip on the magnetic microparticles via the specific binding of biotin and streptavidin. After washing out the uncaptured liposomes, the change in fluorescence intensity before and after heating was measured. As expected, the liposomes with higher lamellarity exhibited a stronger fluorescence signal intensity, as shown in Figure 6C. Biotin-TLip, prepared with a static mixer at an initial lipid concentration of 30, 60, and 90 mg/mL, have lamellarities of 1.4, 4.5, and 7.5, respectively (Table 1). The fluorescence signal intensity of these liposomes was 2.1, 4.7, and 6.7 times stronger than that of unilamellar liposomes prepared by extrusion.

### 3.3 Detection of PSA by using IgG-TLip and IgG-MMP

To verify whether the increased fluorescence signal intensity in the highly lamellar structure of temperature-responsive liposomes is effective in improving the detection sensitivity of biomarkers in TLip-LISA, liposomes with different lamellarity were prepared using the extrusion method and a static mixer. As shown in Table 2, the lamellarity of liposomes prepared using extrusion and a static mixer was 2.6 and 6.5, respectively. The particle size and zeta-potential were comparable for both. Maleimide-PEG lipids were incorporated into the outermost layer of pre-formed TLips, which were then mixed with IgG thiolated with Traut’s reagent was mixed to react with the maleimide-PEG lipids. The amounts of maleimide-PEG lipids and IgG added to the liposome dispersions were adjusted considering that the lipid ratio constituting the outermost layer differs for liposomes with different lamellarity. Therefore, the weight ratio of IgG/SQR22 was 0.14 and 0.06 in IgG-TLip prepared using extrusion and a static mixer, respectively (Table 2).

**TABLE 2:** Characterization of IgG-modified temperature-responsive liposomes (IgG-TLip) prepared by the extrusion method or the microfluidic method using a static mixer.

Preparationmethod	Lamellarity (−)	Particlesize (nm)	PdI	Zeta-potential(mV)	IgG/SQR22 (w/w)
Extrusion (0.2 µm)	2.6	185 ± 54	0.09	−8.7	0.14
Static mixer	6.5	189 ± 70	0.15	−9.4	0.06

**FIGURE 7:**
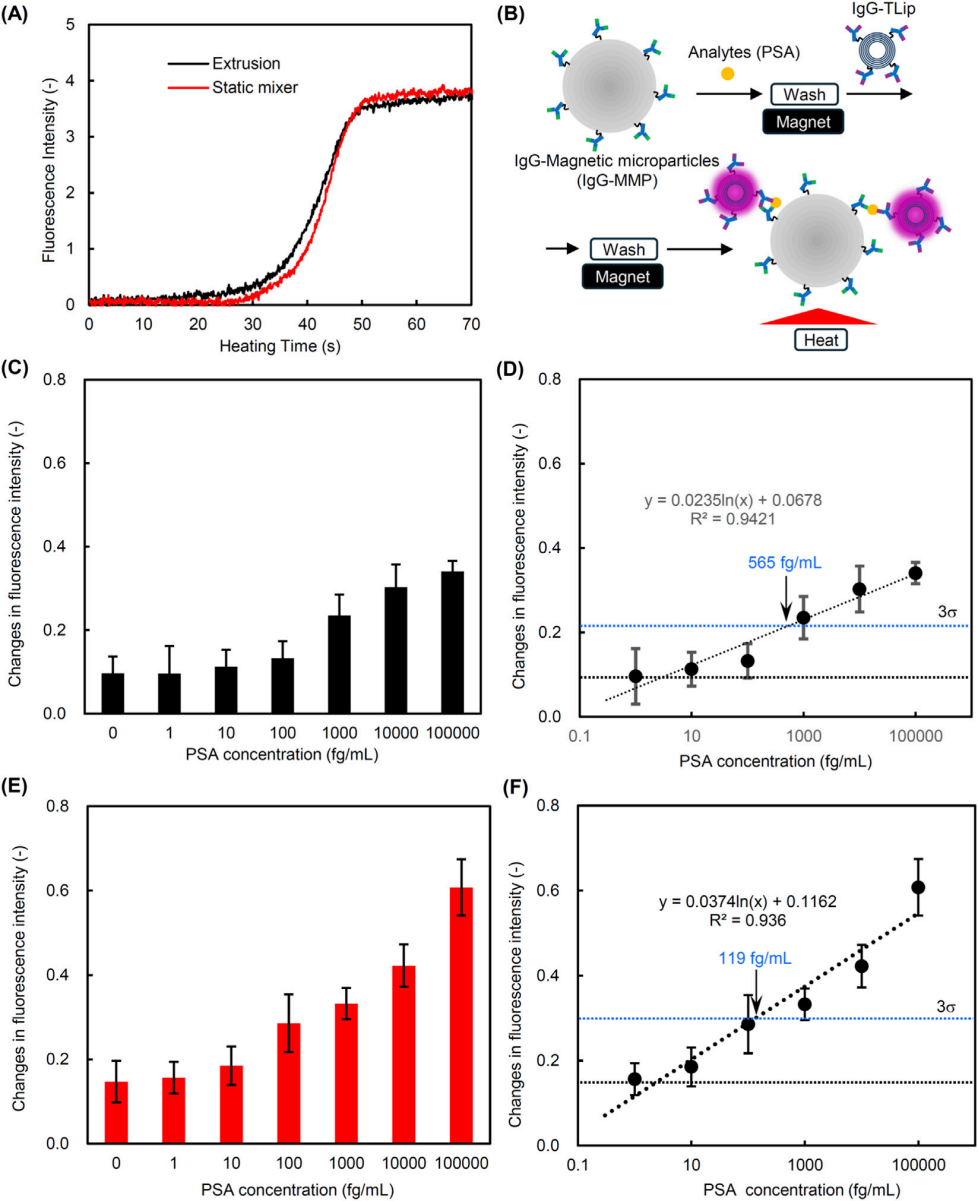
Comparison of PSA detection sensitivity in temperature-responsive liposome-linked immunosorbent assay (TLip-LISA). **(A)** Profiles of fluorescence intensity changes upon heating of IgG-TLip ([lipid] = 0.1 mg/mL) prepared by extrusion or with a static mixer, **(B)** Schematics of TLip-LISA to quantify analytes (PSA) using magnetic microparticles, **(C)** Change in fluorescence signal intensity to detect PSA using IgG-TLip prepared with the extrusion method (n = 3), **(D)** Calibration curve to quantify PSA concentration by using IgG-TLip prepared with the extrusion method, **(E)** Change in fluorescence signal intensity to detect PSA using IgG-TLip prepared with a static mixer (n = 3), and **(F)** Calibration curve to quantify PSA concentration by using IgG-TLip prepared with a static mixer.

Two types of IgG-TLip dispersions exhibited a similar change in fluorescence intensity when heated at the same SQR22 concentration (Figure 7A). The sensitivity of two IgG-TLips for PSA detection was compared. Magnetic microparticles modified with an anti-PSA antibody (IgG-MMP) were prepared for capture of the PSA antigen in an immunosorbent assay. The captured PSA and IgG-TLip were sequentially bound to the IgG-MMP. After washing out the unbound IgG-TLips, the change in fluorescence intensity from the IgG-TLip bound to IgG-MMP via PSA was measured while heating (Figure 7B). With increasing the PSA concentration, the change in fluorescence intensity increased in both IgG-TLips. Comparing the degrees of change in fluorescence intensity at the same PSA concentration revealed that the IgG-TLip prepared with a static mixer (Figure 7E) showed a higher degree than the IgG-TLip prepared by the extrusion method (Figure 7C). To calculate the limit of detection, the degrees of change were plotted against the PSA concentrations. As shown in Figures 7D,F, the limits of detection for PSA were calculated to be 565 fg/mL for the IgG-TLip prepared by the extrusion method and 119 fg/mL for the IgG-TLip prepared with a static mixer, using the 3σ method.

## 4 Discussion

Based on the hypothesis that a multilamellar structure enhances signal amplification in liposome-based biosensing applications and improves the detection sensitivity, we investigated the conditions required to control the lamellarity of size-controlled liposomes using a microfluidic method with a static mixer. Since the flow velocity can be adjusted by altering the flow rate and channel diameter, we used two types of static mixers with different channel diameter (0.6 mm or 1 mm) to examine a broad range of flow velocities. Compared to the conventional microfluidic devices, such as the staggered herringbone mixer (channel depth: 0.08 mm; channel width: 0.2 mm), which are generally used to prepare liposomes or lipid nanoparticles (Chen et al., 2012; Kastner et al., 2015; Cheung and Al-Jamal, 2019; 2019; Ota et al., 2023), the static mixer has a larger flow channel. Flowing fluids in the channel are effectively mixed through a three-dimensional chaotic flow, which consists of the fluids being divided, converted, and inverted by the repeated units of elements placed in the channel of the static mixer (Hobbs and Muzzio, 1997; Thakur et al., 2003). There was no significant difference in the size and lamellarity between liposomes prepared using static mixers with a channel diameter of 0.6 mm or 1 mm when compared at the same TFV (Figure 2). This suggests that the mixing rate and efficiency are equal regardless of channel size if the flow velocity is the same. On the other hand, the TFV significantly affected the particle size and lamellarity of liposomes.

The FRR of aqueous and lipid/ethanol solutions is one of the controllable microfluidic parameters (Jahn et al., 2007; Cheung and Al-Jamal, 2019; Akar et al., 2024). In the FRR of 2–9, the impact of FRR on the particle size was minor regardless of ILC and TFV (Figures 3A–C). Cheung and Al-Jamal (2019) also observed that the effect of FRR on the particle size of liposomes prepared by a microfluidic method using a staggered herringbone mixer was minor at FRR of 3 or above (Cheung and Al-Jamal, 2019). They also indicated that increasing the FRR in the range of 1 and 3 resulted in a decrease in liposome size. We observed that the average size of liposomes prepared at the FRR of 2 was slightly larger than that at the FRR of 3 when the TVF is 47.2 cm/s. However, the change in size is not large, and this trend was not observed at the lower TVFs. The effect of the FRR was also minimal on the lamellarity when the TFV was 8.8 cm/s or more at all ILCs (Figures 3G,H). On the other hand, the lamellarity showed a maximum at the FRR of 3 when the TFV was 3.2 cm/s and the ILC was 60 (Figure 3H) and 90 mg/mL (Figure 3I). This tendency in lamellarity variation was more pronounced at 90 mg/mL than at 60 mg/mL. These results indicated that the FRR affected the lamellarity of liposomes at specific conditions of TFV and ILC, in which an FRR of 3 is appropriate for varying lamellarity over a wide range. Therefore, the FRR was fixed at 3 in the following experiments, and liposomes prepared over a wider range of TFV and ILC were analyzed in their size and lamellarity.

The TFV (3.2–70.7 cm/s) and ILC (30–120 mg/mL) showed an obvious correlation with the particle size and lamellarity of the prepared liposomes (Figure 4). The effect of ILC on the lamellarity was most pronounced at the lowest TFV of 3.2 cm/s (Figures 4C, F). Our data indicated that the lamellarity was largely decreased when the TFV was increased to 8.8 cm/s or more at all ILCs. The liposome formation in the ethanol injection method is known to occur via the formation of self-assembled intermediate structures known as bilayer phospholipid fragments (BPFs) by lipids during mixing of the ethanol and aqueous phases (Lasic and Martin, 1990; Gouda et al., 2021). It is estimated that the formation of BPFs begins at the 80% ethanol condition, and the BPFs transfer to a closed vesicular structure (liposomes or lipid nanoparticles) at the 60% ethanol condition (Maeki et al., 2017). In this mechanism, the size of the liposome can be controlled by the size of the BPFs, and the size of the BPFs can be controlled by the residence time of lipids at the ethanol concentrations of 60%–80%. In microfluidic mixing, TFV or TFR is a factor to control the mixing rate in the channels. At high TFV, rapid mixing leads to the formation of small BPFs followed by the formation of small unilamellar liposomes. On the other hand, low TFV leads to the formation of larger BPF and multilamellar liposomes. In addition, higher ILC at low TFV facilitates the formation of multilamellar liposomes. Typically, the range for the TFV in preparation of liposomes with conventional microfluidic devices is more than 10 cm/s (Hood et al., 2013). To obtain multilayer structures using microfluidic methods, lower TFV needs to be explored (Figure 4C). A lower TFV might cause a low volumetric throughput in conventional microfluidic devices due to the narrow flow channels. Static mixers have the advantage that the channel diameter can be changed, allowing the high volumetric throughput and mixing efficiency to be maintained even at a low TFV.

The mean diameter of liposomes with the maximum lamellarity of 20.5 was 324 nm (Figure 4H). For these liposomes, the estimated geometric maximum thickness of the multilayered membrane is 162 nm, assuming the inner space of the liposomes is filled with a lamellar membrane. While the presence of an inner aqueous phase may need to be considered for multilamellar liposomes, because the lower limit size of DPPC vesicles is estimated to be 20 nm in diameter (Cornell et al., 1982; Marrink and Mark, 2003). In a multilamellar liposome model with an innermost layer of 20 nm in diameter, the lamellar thickness of a 324 nm liposome is calculated to be 156 nm. Based on the repeat spacing (6.4 nm) of the DPPC multilamellar membrane (Bryant et al., 2019), which includes the bilayer thickness and the water layer between them, the number of layers to form a 156 nm-thick multilayered membrane is calculated to be 24. This value represents the maximum theoretical lamellarity, as estimated from a geometric model of a typical 324 nm multilamellar liposome. It should be noted that there are limitations in the comparative analysis between the theoretical estimation and experimental measurement of the lamellarity of liposomes because actual liposome samples involve distribution in particle size and structural diversity (e.g., multilamellar vesicles, multivesicular vesicles, and their hybrids) (Guimarães et al., 2021). Additionally, in this study, the lamellarity of actual liposomes was calculated from the relative surface area of liposomes under the assumption that the number of lipids composing each lipid layer is the same as the number of lipids composing the outermost layer of liposomes. Therefore, the lamellarity should be proportional to the fluorescence intensity of SQR22 per liposome, as its value represents the relative amount of lipids containing SQR22 per liposome. Theoretically, the inner layers should have smaller diameters and contain fewer lipids than the outermost layer. The influence of the difference in the number of lipids in each layer becomes more pronounced as the lamellarity increases. Further research is needed on the detailed analysis of the internal structure of liposomes and on constructing a calculation model that accounts for differences in the number of lipids in each layer based on the actual lamellar structure and size distribution to enhance the reliability of lamellarity estimation and measurement for liposomes or lipid nanoparticles with internal lipid molecular assemblies.

TLip based on the phase transition of DPPC membrane are in a gel state at temperatures below the phase transition temperature (around 41 °C). The colloidal dispersion stability of DPPC liposomes with a neutral surface is low, and aggregation between liposomes is often observed, especially in the gel phase. Nonspecific liposome aggregation would interfere with quantification in biosensing applications. Adding a negatively charged component (DHSG) to liposomes prevents liposome aggregation through electrostatic repulsion between liposomes. Furthermore, the negatively charged surface prevents nonspecific interactions with proteins, which cause the aggregation and destabilization of liposomes, because most proteins are negatively charged. From this viewpoint of colloidal stability and interactivity, negatively charged liposomes, which have high dispersion stability and low nonspecific reactivity with biological components, have an advantage in biosensing applications. On the other hand, adding negatively charged lipids such as DHSG is an effective way to reduce the lamellarity of liposomes due to electrostatic repulsion between lamellar membranes in a top-down approach for liposome preparation using conventional batch methods such as extrusion (Takeoka et al., 1996; Sou, 2011). Therefore, the formation of highly multilayered charged liposomes may be a notable feature of the microfluidic method, which differs from the conventional batch methods. Although further research is required to explain the detailed mechanism of formation of multilamellar liposomes with a microfluidic method using a static mixer, slow mixing at low TFV may lead to the layering of BPFs, specifically at higher ILC. Controlling the structure of lipid molecular assemblies is becoming increasingly important for controlling not only particle size but also internal structure. In the case of lipid nanoparticles encapsulating nucleic acid molecules, highly ordered internal structures have been observed (Viger-Gravel et al., 2018; Udepurkar et al., 2025). The microfluidic method using a static mixer is expected to apply not only to the control of the size and lamellarity of liposomes but also to the structural control of lipid nanoparticles.

As shown in Figure 5, the lamellarity of liposomes is decreased by increasing the processing temperature. Although lower temperatures are desirable for preparing highly multilamellar liposomes, the formation of lipid aggregates was observed when TLip, which exhibits gel-to-liquid crystalline phase transition at 41 °C, were prepared below the transition temperature. A part of lipids would solidify and aggregate before forming liposomes because the hydration of lipids takes a longer time in a gel state in general. Lipid hydration is facilitated by heating above the phase transition temperature of the liposomes. Therefore, the processing temperature through the static mixer was set to 50–55 °C. The FRR of 3 and the TFV of 3.2 cm/s were applied for the preparation of TLip. Biotin-TLip with different lamellarities were successfully prepared by changing the ILC (Table 1). Although the profiles of the fluorescence intensity change of Biotin-TLip upon heating were the same for liposomes with different lamellarities (Figure 6B), a clear difference in the degree of change in fluorescence intensity was observed when capturing the Biotin-TLip with streptavidin-modified magnetic microparticles (Figure 6C). The rate of increase in fluorescence intensity was almost proportional to the rate of increase in lamellarity, supporting that the difference in fluorescence signal reflects the difference in the lamellarity rather than the difference in the number of liposomes bound to the magnetic microparticles.

To verify whether differences in fluorescence intensity due to lamellarity of liposomes improve the detection sensitivity of biomarkers, two types of IgG-TLip with different lamellarity were prepared by the extrusion method and the microfluidic method. We attempted to obtain IgG-TLip of the same size but with different lamellarity using a static mixer, compared with IgG-TLip (185 ± 54 nm) prepared by the conventional extrusion method. IgG-TLip of equal size (189 ± 70 nm) could be successfully prepared under the condition of TRR of 3, TRV of 3.2 cm/s, and ILC of 90 mg/mL (Table 2). As mentioned in the previous paragraph, the preparation of TLip required heating to 50–55 °C to facilitate lipid hydration. Increased temperature causes the lower lamellarity of liposomes (Figure 5). This is the reason why the lamellarity of IgG-TLip is lower than that of liposomes containing cholesterol (Figure 4H), which were prepared at room temperature. We adopted a post-modification approach to modify the surface of pre-formed TLip by maleimide-PEG lipid and antibodies. Although PEG lipids themselves form micelles in the aqueous media, the hydrophobic part of the PEG lipid is spontaneously incorporated into the outer layer of lipid bilayer membrane of liposomes when they are mixed with pre-formed liposomes, resulting in a surface modification of liposomes with PEG (Sou et al., 2000). The reason for adopting post-modification is to eliminate the possibility that PEG existing between lipid bilayers of lamellar structure would result in repulsion between the lipid bilayers, which would act to reduce the lamellarity of liposomes. Furthermore, this method can reduce the amount of surface modifiers required because only the outer surface of the liposome is modified. The antibody conjugation efficiency, which is calculated from the weight ratio of antibody to lipids constituting the outermost layer of TLip in the reaction mixture and resulting IgG-modified TLip after removing the unconjugated IgG, was 5.7% for TLip (lamellarity of 6.5) prepared by a static mixer and 5.4% for TLip (lamellarity of 2.6) prepared by extrusion (Supplementary Table S2). This result indicates that differences in lamellarity have little effect on antibody binding efficiency. The weight ratio of IgG/SQR22 was 0.14 and 0.06 in IgG-TLip prepared using extrusion and a static mixer, respectively (Table 2). Although the amount of IgG is different between IgG-TLips with different lamellarity, the IgG/SQR22 ratio in the outermost layer of liposomes is comparable when considering their lamellarity.

As shown in Figures 7C,E, the change in fluorescence intensity using IgG-TLip prepared using a static mixer was larger than that using IgG-TLip prepared by extrusion when compared at the same PSA concentration. The calculated limit of detection was 119 fg/mL and 565 fg/mL for IgG-TLip prepared by a static mixer and extrusion, respectively, supporting that the increasing lamellarity of IgG-TLip is effective in increasing the detection sensitivity. The detection sensitivity in the previous TLip-LISA that utilized the time difference of temperature-responsive fluorescence emission between TLip binding to the bottom of a 96-well plate via targets and unbinding TLip was as high as 27.6 ag/mL for PSA (Hu et al., 2020). This conventional format has the advantage that the fluorescence of TLip can be measured without washing, and one of the reasons for its high sensitivity is that there is no loss of binding TLip during the washing process. However, the dynamic range of the calibration curve for quantification was limited between 10 ag/mL to 1 fg/mL. The current format using IgG-TLip and magnetic microparticles requires a washing process to remove unbound IgG-TLip before fluorescence measurement. The loss of bound IgG-TLip during this washing step can be a possible reason for the lower sensitivity compared to the previous TLip-LISA without washing. On the other hand, the current format using magnetic microparticles has the advantage that it can be adapted to automation using a flow system incorporating separation of magnetic microparticles using a magnet. Additionally, the calibration curve correlates with PSA concentrations in the range of 1 fg/mL to 100 pg/mL. Compared to the detection limit for PSA in general ELISA (10–100 pg/mL), the current format using multilamellar liposomes has a detection limit that is 2–3 orders of magnitude lower.

## 5 Conclusion

The microfluidic method using a static mixer is useful for preparing size- and lamellarity-controlled liposomes. The size and lamellarity of liposomes can be controlled by altering the microfluidic conditions. Specifically, the TFV and the ILC were significant parameters in controlling the lamellarity. Increased lamellarity increases the amount of fluorescent probe molecules that can be loaded in one liposome, resulting in an enhanced fluorescence signal per liposome. As expected, a fluorescent probe based on the liposomes with higher lamellarity exhibited superior biomarker detection sensitivity in an immunosorbent assay. This finding can be applied to improving the sensitivity of liposome-based biosensing and bioimaging. Controlling lipid molecular assembly using microfluidic methods is increasing the importance not only for liposomes, but also for lipid nanoparticles containing nucleic acid molecules. In conventional microfluidic devices, scaling up is achieved through the numbering-up or piling-up of the channel or device unit. The advantage of these approaches is that the microfluidic conditions set by the device unit can be applied to scaling up with minimal modifications. However, an increased number of channels or device units may complicate the flow control. Additionally, microfluidic devices with narrow channels are prone to clogging, which makes cleaning the channels difficult and costly. The microfluidic method using a static mixer can be scaled by changing the size of the flow channel and element. Although further investigation is required over a wider range of channels, this study suggested that comparable liposomes in size and lamellarity can be obtained regardless of the channel size if the total flow velocity is the same. Static mixers would facilitate the continuous flow manufacturing of structurally controlled liposomes and lipid nanoparticles for biosensing and other therapeutic applications.

## Data Availability

The original contributions presented in the study are included in the article/Supplementary Material, further inquiries can be directed to the corresponding authors.
