# Supplementary material for: Microfluidic preparation of size- and lamellarity-controlled liposomes using a static mixer and the advantage of a multilamellar structure in biosensing applications
Source: Front Bioeng Biotechnol. 2026 Jan 7;13:1715496. doi: 10.3389/fbioe.2025.1715496 (PMC12819795; doi:10.3389/fbioe.2025.1715496)
Supplement: Supplementary file 1 [file Supplementaryfile1.docx]

Supplementary Material

Microfluidic Preparation of Size- and Lamellarity-controlled Liposomes Using a Static Mixer and the Advantage of a Multilamellar Structure in Biosensing Applications

Makoto Kiuchi^1^, Shinji Takeoka^1,2*^, Keitaro Sou^2*^

^1^ Department of Life Science and Medical Bioscience, Graduate School of Advanced Science and Engineering, Waseda University, Tokyo, Japan.

^2^Waseda Research Institute for Science and Engineering, Waseda University, Tokyo, Japan

*** Correspondence:**Shinji Takeoka
takeoka@waseda.jp

# Keitaro Sou soukei@aoni.waseda.jp

1. **Supplementary data for the measurement and calculation of lamellarity**


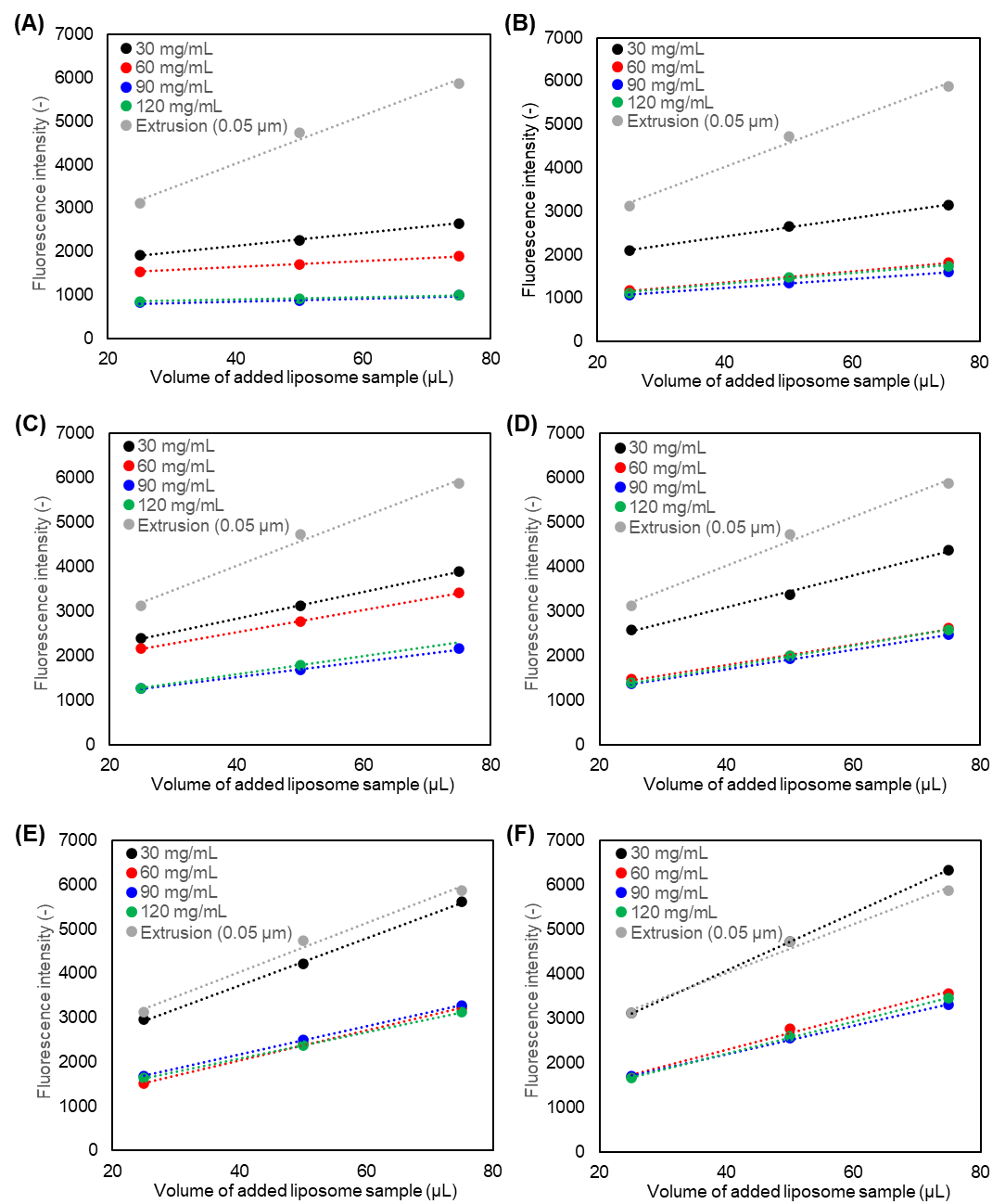


**Figure S1.** The plots of added volume of liposome samples and fluorescence intensity of 6-p-toluidino-2-naphthalenesulfonic acid (TNS) for determining the number of bilayer membranes (lamellarity) of liposomes. Liposomes were prepared using a static mixer at various total flow velocities (TFVs) with a flow rate ratio (FRR) of 3 and initial lipid concentrations (ILCs) of 30, 60, 90, and 120 mg/mL. (A) TFV of 3.2 cm/s, (B) TFV of 8.8 cm/s, (C) TFV of 17.0 cm/s, (D) TFV of 25.5 cm/s, (E) TFV of 47.2 cm/s, and (F) TFV of 70.7 cm/s.

**Table S1.** Calculation of the lamellarity of liposomes prepared using a static mixer under various conditions.

| **Total flow velocity (TFV) (cm/s)** | **Initial lipid concentration (ILC) (mg/mL)** | **Slope^1^  (*S_b_*)** | | **Lamellarity^2)^** |
| --- | --- | --- | --- | --- |
| 3.2 | 30 | 14.74 | 3.7 | |
|  | 60 | 7.16 | 7.7 | |
|  | 90 | 3.45 | 16.0 | |
|  | 120 | 2.69 | 20.5 | |
| 8.8 | 30 | 20.88 | 2.6 | |
|  | 60 | 12.98 | 4.3 | |
|  | 90 | 10.35 | 5.3 | |
|  | 120 | 12.40 | 4.5 | |
| 17.0 | 30 | 29.85 | 1.9 | |
|  | 60 | 25.04 | 2.2 | |
|  | 90 | 17.65 | 3.1 | |
|  | 120 | 17.41 | 3.2 | |
| 25.5 | 30 | 35.75 | 1.5 | |
|  | 60 | 23.08 | 2.4 | |
|  | 90 | 22.25 | 2.5 | |
|  | 120 | 23.92 | 2.3 | |
| 47.2 | 30 | 53.44 | 1.0 | |
|  | 60 | 34.03 | 1.6 | |
|  | 90 | 31.97 | 1.7 | |
|  | 120 | 29.87 | 1.8 | |
| 70.7 | 30 | 64.49 | 0.9 | |
|  | 60 | 37.69 | 1.5 | |
|  | 90 | 32.20 | 1.7 | |
|  | 120 | 35.73 | 1.5 | |
| Extrusion (0.05 µm) |  | (*S_a_*) 55.23 | 1.0 | |

1. The slope was calculated from a least-squares fit of the plots shown in Figure S1.
2. Lamellarity was calculated by dividing the slope of the unilamellar liposomes prepared by extrusion (final filter pore of 0.05µm) (*S_a_*) by the slope of liposome samples prepared using a static mixer (*S_b_*) from equation (1). *Lamellarity*=*S_a_*/*S_b_* (1)

**
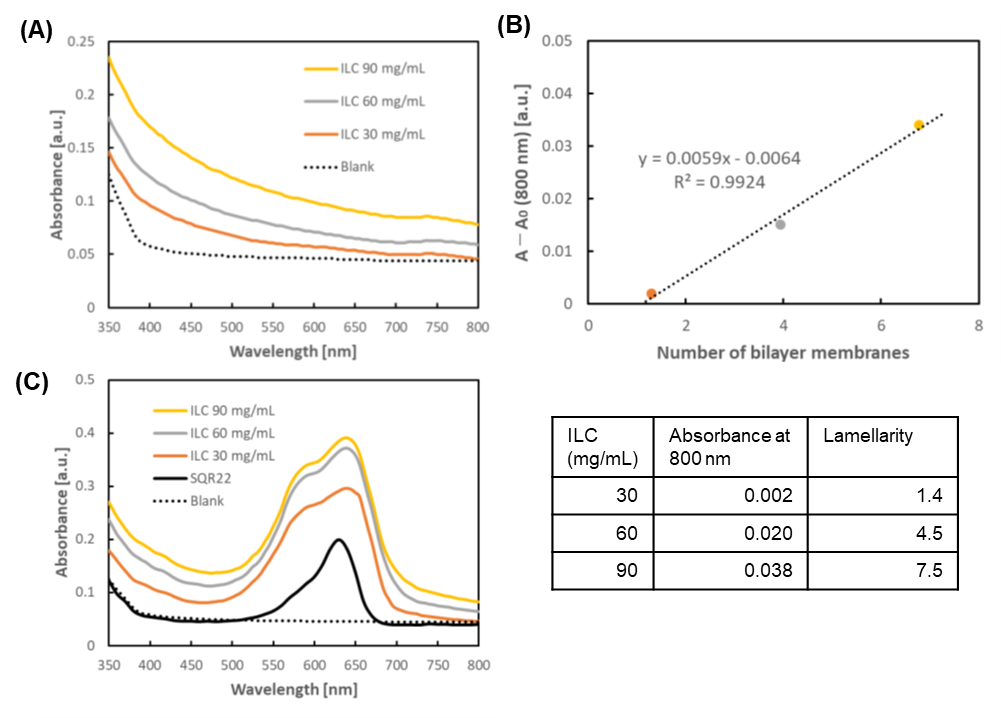
**

**Figure S2.** Measurement of the lamellarity of temperature-responsive liposomes containing SQR22. (A) UV-vis spectra of temperature-responsive liposomes (without SQR22) dispersion prepared using a static mixer under a flow rate ratio (FRR) of 3, a total flow velocity (TFV) of 3.2 cm/s, and initial lipid concentrations (ILCs) of 30, 60, and 90 mg/mL. Lipid concentration was adjusted to be 0.5 mg/mL before measurement. (B) Calibration curve to determine the lamellarity from the absorbance (turbidity) at 800 nm ([Lipids]=0.5 mg/mL). The number of bilayer membranes (lamellarity) was measured by the same method using 6-p-toluidino-2-naphthalenesulfonic acid (TNS) shown in Figure S1 and Table S1. (C) UV-vis spectra of temperature-responsive liposomes (with SQR22) dispersion. Absorbance at 800 nm was used to determine the lamellarity from the calibration curve shown in Figure S2B. The spectra of SQR22 indicate that absorption by SQR22 does not interfere with the absorbance at 800 nm.

1. **Supplementary data for calculation of IgG conjugation efficiency**

**Table S2.**  IgG conjugation efficiency to maleimide-modified temperature-responsive liposomes (Mal-TLip) prepared by the extrusion method or the microfluidic method using a static mixer.

| **Preparation method** | **Lamellarity (-)** | **IgG/lipids (outer layer)**  **in the reaction mixture**  **(w/w)** | **IgG/lipids (outer layer)**  **in the purified IgG-TLip**  **(w/w)** | **IgG conjugation efficiency (%)** |
| --- | --- | --- | --- | --- |
| Extrusion (0.2 µm) | 2.6 | 0.07 | 3.78×10^-3^ | 5.4 |
| Static mixer | 6.5 | 0.07 | 4.07×10^-3^ | 5.7 |

## Preparation and characterization of IgG-conjugated magnetic microparticles (IgG-MMP)

Anti-human PSA antibody-conjugated magnetic microparticles were prepared via chemical crosslinking between the amino group of the capture antibody and the carboxyl group on the magnetic microparticles (Magnosphere™ MS300/Carboxyl, Carboxyl-MMP). The carboxyl-MMP dispersion (1 mL, [solid]=10 mg/mL) was transferred to a microtube. The microtube was placed on a magnetic stand for 1 minute, and then the supernatant was removed. Then, the reaction buffer (0.1 M 2-(*N*-morpholino) ethanesulfonic acid buffer, pH 5.0, 1 mL) was added to the microtube, and the Carboxyl-MMP was dispersed using a vortex mixer. The coupling agent, 1-ethyl-3-[3-dimethylaminopropyl] carbodiimide (EDAC), dissolved in the same reaction buffer (10 mg/mL, 100 µL), was added to the Carboxyl-MMP dispersion. The mixture was gently shaken for 30 minutes at room temperature. Then, the anti-human PSA antibody (clone: 4D10, 99.9 µg) was added to the reaction mixture. The mixture was shaken gently for 3 hours at room temperature. After the reaction, the microtube was placed on a magnetic stand to remove the supernatant. Then, washing buffer (25 mM Tris-HCl, pH 7.2, 0.15 M NaCl, 0.05 % Tween 20) was added to the microtube, and the antibody-conjugated magnetic microparticles were dispersed using a vortex mixer. The microtube was placed on a magnetic stand, and the supernatant was removed again to wash the antibody-conjugated magnetic microparticles. This washing cycle was repeated three times. Finally, the antibody-conjugated magnetic microparticles (IgG-MMP) were dispersed in 1 mL of DPBS (0.02% NaN_3_) and stored in a refrigerator.

The size distribution, mean diameter, and zeta potential of the prepared IgG-MMP were measured by the Zetasizer Nano S90 (Malvern Instruments Ltd., Malvern, UK). The amount of antibody that bound to the magnetic microparticles was determined by quantifying the amount of unbound antibody in the supernatant after the reaction using a fluorescamine assay as described above. The characterizations are summarized in Table S3.

**Table S3.**  Characterization of anti-human PSA antibody-conjugated magnetic microparticles (IgG-MMP).

| **Samples** | **Particle size (nm)** | **PdI** | **Zeta potential (mV)** | **IgG concentration (µg/mL) in IgG-MMP dispersion ([solid]=10 mg/mL)** |
| --- | --- | --- | --- | --- |
| Carboxyl-MMP | 2423 | 0.498 | -40.5±2.2 | - |
| IgG-MMP | 2777 | 0.446 | -12.1±2.0 | 63.1 |
